# Chitosan and Hyaluronic Acid Nanoparticles as Vehicles of Epoetin Beta for Subconjunctival Ocular Delivery

**DOI:** 10.3390/md20020151

**Published:** 2022-02-18

**Authors:** Beatriz Silva, Lídia M. Gonçalves, Berta São Braz, Esmeralda Delgado

**Affiliations:** 1 CIISA—Centre for Interdisciplinary Research in Animal Health, Faculty of Veterinary Medicine, University of Lisbon, Avenida da Universidade Técnica, 1300-477 Lisbon, Portugal; beatrizsilva@fmv.ulisboa.pt (B.S.); esmeralda@fmv.ulisboa.pt (E.D.); 2 Associate Laboratory for Animal and Veterinary Sciences (AL4AnimalS), 1300-477 Lisbon, Portugal; 3 Research Institute for Medicines (iMed.ULisboa), Faculty of Pharmacy, University of Lisbon, 1600-277 Lisbon, Portugal; lgoncalves@ff.ulisboa.pt

**Keywords:** nanoparticles, mucoadhesion, chitosan, hyaluronic acid, erythropoietin, epoetin beta, ocular delivery

## Abstract

Neuroprotection in glaucoma using epoetin beta (EPOβ) has yielded promising results. Our team has developed chitosan-hyaluronic acid nanoparticles (CS/HA) designed to carry EPOβ into the ocular globe, improving the drug’s mucoadhesion and retention time on the ocular surface to increase its bioavailability. In the present in vivo study, we explored the possibility of delivering EPOβ to the eye through subconjunctival administration of chitosan-hyaluronic acid-EPOβ (CS/HA-EPOβ) nanoparticles. Healthy Wistar Hannover rats (*n* = 21) were split into 7 groups and underwent complete ophthalmological examinations, including electroretinography and microhematocrit evaluations before and after the subconjunctival administrations. CS/HA-EPOβ nanoparticles were administered to the right eye (OD), and the contralateral eye (OS) served as control. At selected timepoints, animals from each group (*n* = 3) were euthanized, and both eyes were enucleated for histological evaluation (immunofluorescence and HE). No adverse ocular signs, no changes in the microhematocrits (≈45%), and no deviations in the electroretinographies in both photopic and scotopic exams were observed after the administrations (*p* < 0.05). Intraocular pressure remained in the physiological range during the assays (11–22 mmHg). EPOβ was detected in the retina by immunofluorescence 12 h after the subconjunctival administration and remained detectable until day 21. We concluded that CS/HA nanoparticles could efficiently deliver EPOβ into the retina, and this alternative was considered biologically safe. This nanoformulation could be a promising tool for treating retinopathies, namely optic nerve degeneration associated with glaucoma.

## 1. Introduction

Neurodegenerative ocular diseases, such as glaucoma, have a major impact on people’s daily life, and the interest in neuroprotection as part of glaucoma treatment has been increasing [1]. Erythropoietin (EPO) is a glycoprotein produced in adult kidneys and fetal liver that has a primary hematopoietic role of stimulating the production of erythrocytes in the bone marrow [2]. However, novel EPO secreting sites were discovered, like the brain [3] and the retina [4], which have also demonstrated the expression of EPO receptors [5,6]. Recent studies demonstrated that EPO has tissue-protective properties in the brain, heart, inner ear, and retina [7], and the subconjunctival administration of EPO, as an alternative delivery method aiming at the posterior ocular segment, has been explored with promising results [8,9]. Recent investigation with glaucomatous rats demonstrated that epoetin beta (EPOβ), a recombinant version of EPO, administered by subconjunctival route, reached the retina and improved its condition when compared to non-treated animals [10].

Topical administration is considered ineffective for the treatment of posterior ocular diseases; systemic administration may result in severe side effects, and intravitreal injection is invasive and may lead to side effects like increased intraocular pressure, vitreous detachment, retinal hemorrhage/ toxicity, and cataracts [11]. Subconjunctival administration is a minor-invasive procedure with few side effects and it is considered a promising alternative route of administration for the treatment of retinal diseases [12]. Nevertheless, despite enabling a sustained drug delivery, a percentage of the drug is always cleared out by conjunctival blood or lymphatic flow [13]. Thus, it is important to improve the drug’s retention time and penetration through the outer ocular layers to increase its bioavailability when administered subconjunctivally.

Mucoadhesive nanoparticles are state-of-the-art systems that can fulfill those requirements by protecting the drug while enhancing its permeation across biological barriers [14]. Chitosan is a well-studied natural polymer with excellent mucoadhesive characteristics. Its cationic nature enables ionic interactions with the ocular mucosa (negatively charged), which improve the drug’s mucoadhesion and retention time on the ocular surface [15,16]. Therefore, chitosan nanoparticles can reduce the frequency of ocular administrations and improve long-term patient compliance [17]. Chitosan also acts like a permeation enhancer by widening the tight junctions of the cell membranes [18]. Moreover, it has a low production cost and a reduced ecological impact because it is obtained from crustaceans exoskeletons and fungi cell walls by deacetylation of chitin [19]. A recent study used chitosan-coated nanoparticles for the delivery of bevacizumab to the posterior segment of the eye by subconjunctival administration, and the results showed higher drug concentration in the posterior segment and better results in the retinopathy model, compared to the topical and intravitreal administrations [20]. Chitosan can be associated with various synthetic and natural compounds, such as hyaluronic acid. Hyaluronic acid is a natural polysaccharide distributed throughout ocular tissues, such as the vitreous body, lacrimal gland, human tears, conjunctiva, and corneal epithelium [21]. When considering ophthalmic formulations, their mucins-like viscoelastic and biophysical properties and their additional mucoadhesive strength due to CD44 receptor-mediated binding are valuable features [22,23].

Recently, our team has developed a system of chitosan-hyaluronic acid nanoparticles (CS/HA) designed to carry EPOβ into the ocular globe. In vitro and ex vivo testing of the physicochemical stability, cytotoxicity, and mucoadhesive strength of the chitosan-hyaluronic acid-epoetin beta (CS/HA-EPOβ) nanoparticles have been previously performed [24]. Therefore, in the present study, we aimed to evaluate the behavior of the CS/HA-EPOβ nanoparticulate system in vivo. For that, we used Wistar Hanover rats, and we tested the subconjunctival route of administration under physiological conditions. We intended to assess the formulation’s biological tolerance and safety, its local and systemic impact, its influence in retinal electrophysiology, and EPOβ’s distribution in ocular tissues by immunofluorescence.

## 2. Results

### 2.1. Ophthalmological Examinations

On day 1 after the subconjunctival administration, a discrete reddish discharge in the ocular medial canthus of both eyes was observed in 3 rats (one from group B and two from group C), but apart from that, no other ocular changes were detected, namely conjunctival hyperemia, corneal edema or aqueous flare. Therefore, ophthalmological examinations of both eyes were considered normal for all animals (*n* = 21). Moreover, animals exhibited a completely normal behavior and showed no signs of pain or discomfort throughout the entire study.

**Figure 1:**
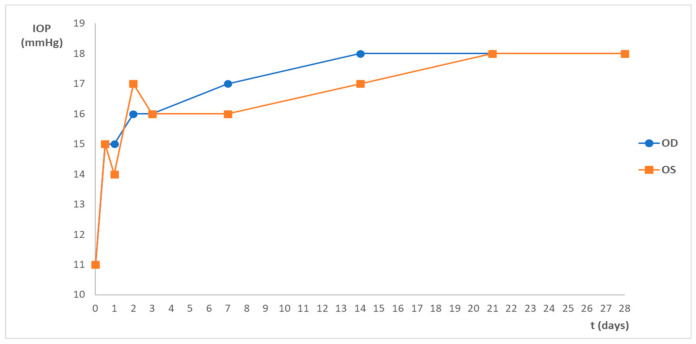
Mean IOP (mmHg) variation during the study after the subconjunctival administration of the nanoparticles. Data represents all groups.

Before the subconjunctival administration, the mean intraocular pressure (IOP) was 17 ± 2 mmHg for the OD and 18 ± 3 mmHg for the OS when the animals were awake. Immediately after the subconjunctival administration (day 0; Figure 1), when animals were still anesthetized, the mean IOP was 11 ± 2 mmHg for the OD and 11 ± 1 mmHg for the OS. The mean IOP of the OD was not significantly different from the OS (control) within groups (*p* < 0.05), but it was different between groups when comparing day 0 with the following timepoints (*p* > 0.05). Nevertheless, the mean IOP of both eyes remained within the physiological range during the whole study, with a minimum IOP of 11 mmHg and a maximum of 22 mmHg (Figure 1).

### 2.2. Hematocrit

The hematocrit of rats was not affected by the subconjunctival administration of the nanoformulation. Considering all animals, the mean microhematocrit before the nanoparticles administration was 44.3 ± 3.1%, and after the administration (before euthanasia), it was 44.8 ± 3.6%. As shown in Table 1, no statistically significant differences were detected in hematocrit values before and after subconjunctival administration (*p* < 0.05), so no significant influence in erythropoiesis was observed as an EPOβ side effect. There were significant statistical differences between groups (*p* > 0.05), which were considered physiological variability as the results were within reference values.

### 2.3. Electroretinography

**Figure 2:**
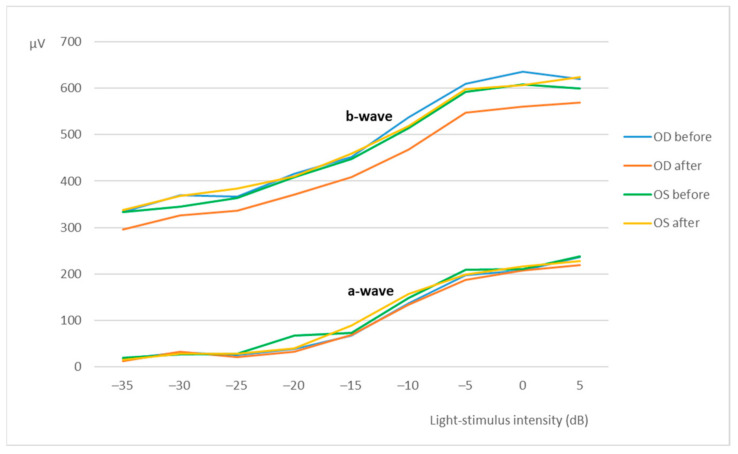
Scotopic luminesce response-mean amplitudes of the a-wave and the b-wave (µV) recorded from the OD and OS in response to increscent light-stimulus intensities (dB). Values correspond to before and after the administration of the nanoparticles (after = before euthanasia). Data represent all groups.

**Figure 3:**
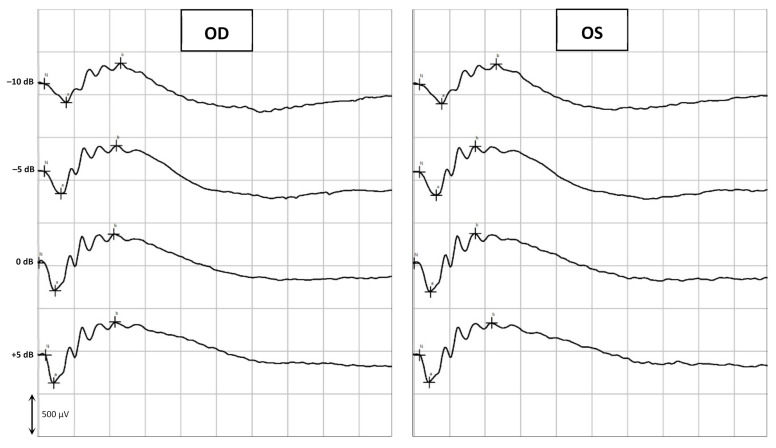
Example of the scotopic luminesce response. The retinal response was recorded from the OD and OS of a rat (group C) before euthanasia in response to crescent light-stimulus intensities (in this example, from –10 dB to +5 dB).

Assessment of the retinal function was achieved by flash electroretinography (ERG), in which the curves and the amplitudes of the a-wave and the b-wave (mean ± SD [min; max]) were analyzed. All groups (from A to F) showed a similar retinal response in both eyes (OU) before and after the subconjunctival administration of the CS/HA-EPOβ nanoparticles. Also, there were no significant differences between the OD and the OS in the different parts of the ERG exam. At the end of the scotopic luminescence response (SLR) with 5 dB of light intensity, before the CS/HA-EPOβ nanoparticles administration, the mean a-wave of the OD was 236 ± 75 [110; 375] μV and the b-wave was 619 ± 147 [379; 1010] μV. After the administration, the OD showed a mean a-wave of 219 ± 62 [110; 311] μV and a mean b-wave of 569 ± 119 [349; 831] μV. No statistically significant differences (*p* < 0.05) were found between the mean amplitudes of the a and b waves of the OD before and after the administration. Concerning the OS, again no significant differences (*p* < 0.05) were detected before (a-wave: 239 ± 68 [114; 346] μV; b-wave: 600 ± 137 [349; 859] μV) and after (a-wave: 228 ± 61 [114; 323] μV; b-wave: 624 ± 118 [391; 844] μV) the administrations, or between OS and OD, at any timepoint. Figure 2 illustrates the variation of the a and b waves according to light stimuli, and both waves increased their amplitude when the light intensity increased. Figure 3 is a real representation of the SLR.

The photopic adaptation (PA) results are shown in Table 2 and reveal no statistically significant difference (*p* < 0.05) between treated and control eyes and among the groups. At 16 min of light adaptation, the mean wave amplitudes for the OD before the administration was 16 ± 12 [2; 37] μV for the a-wave and 200 ± 40 [135; 264] μV for the b-wave, and after the administration, it was 16 ± 11 [1; 35] μV for the a-wave and 212 ± 44 [107; 294] μV for the b-wave. Regarding the OS, the mean a-wave before administration was 19 ± 14 [3; 39] μV and the b-wave was 201 ± 44 [135; 294] μV, and after the administration, it corresponded to 18 ± 12 [1; 37] μV for the a-wave and to 201 ± 46 [117; 294] μV for the b-wave. Waves did not seem to increase or decrease with light stimuli.

**Figure 4:**
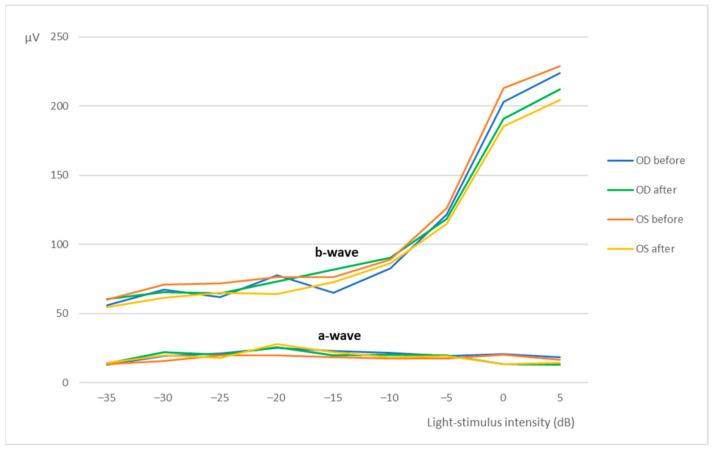
Photopic luminesce response-mean amplitudes of the a-wave and the b-wave (µV) recorded from the OD and OS in response to increscent light-stimulus intensities (dB). Values correspond to before and after the administration of the nanoparticles (after–before euthanasia). Data represent all groups.

The results of the photopic luminescence response (PLR) are illustrated in Figure 4, where the b-wave clearly increases with light intensity, unlike the a-wave, whose amplitudes maintained somewhat constant. Nevertheless, mean amplitudes of a and b waves were similar before and after the administration of the nanoparticles, for the OD and OS, and between OD and OS, in all groups. At 5 dB of light intensity, before the administration of the nanoparticles, the OD presented a-wave of 16 ± 13 [5; 51] μV and b-wave of 224 ± 50 [99; 315] μV, while the OS had a-wave of 16 ± 14 [3; 59] μV and a b-wave of 229 ± 42 [167; 343] μV. After the administration, the a-wave and b-wave were, respectively, 14 ± 6 [3; 29] μV and 212 ± 54 [100; 343] μV for the OD; and 14 ± 15 [3; 60] μV and 205 ± 53 [115; 333] μV for the OS. Thus, no statistically significant differences (*p* < 0.05) were observed among those results.

**Figure 5:**
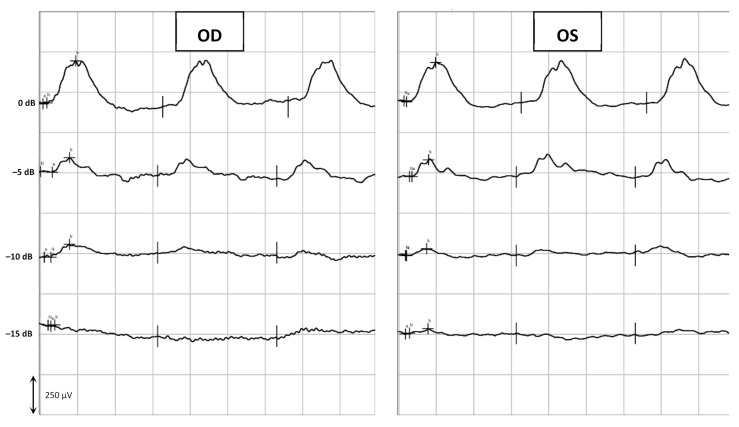
Example of a photopic flicker exam. The retinal response was recorded from the OD and OS of a rat (group D) before euthanasia in response to decrescent light-stimulus intensities (from 0 dB to –15 dB), after 10 min of continuous light exposure.

Photopic flicker (PF) results have a graphic representation in Figure 5. Before the administration of the nanoparticles, after 10 min of light exposure, the a and b waves recorded at 0 dB were, respectively, 6 ± 4 [1; 13] μV and 227 ± 61 [108; 353] μV for the OD; and 8 ± 4 [3; 18] μV and 227 ± 44 [177; 354] μV for the OS. After the administration, the OD had a-wave of 6 ± 3 [1; 12] μV and b-wave of 229 ± 59 [111; 338] μV; and the OS had a-wave of 6 ± 3 [2; 14] μV and 234 ± 43 [139; 318] μV for the OS. Differences in these results were not statistically significant (*p* < 0.05). While the b-wave tends to decrease with the decreasing light stimuli, the a-wave tends to increase.

**Figure 6:**
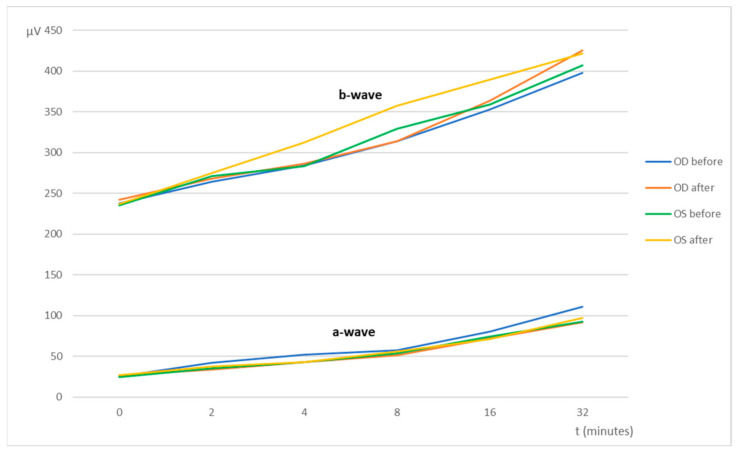
Scotopic Adaptation-mean amplitudes of the a-wave and the b-wave (µV) recorded from the OD and OS in response to light-stimulus after t minutes of dark adaptation (from 0 to 32 min). Values correspond to before and after the administration of the nanoparticles (after–before euthanasia). Data represent all groups.

**Figure 7:**
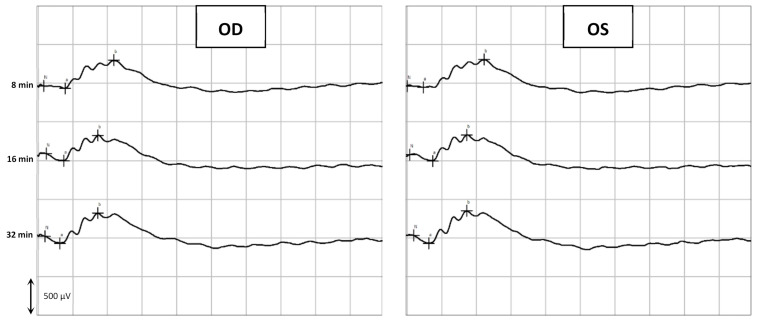
Example of a Scotopic Adaptation exam. The retinal response was recorded from the OD and the OS of a rat (group E) before euthanasia in response to light-stimulus after 0 to 32 min of dark adaptation. This figure shows timepoints 8, 16, and 32 min.

The last part of the ERG exam was the scotopic adaptation (SA), whose results are presented in Figure 6, while Figure 7 shows an example of a SA curve. The longer the dark adaptation, the more the retina appears to respond to light stimuli, as both a and b waves show a tendency to increase with time. That behavior was observed in all groups. At the end of the SA (32 min), the OD before the administration had a mean a-wave of 111 ± 33 [53; 170] μV and a b-wave of 398 ± 60 [303; 514] μV; while after the administration, the a and b waves of the OD were 92 ± 37 [43; 145] μV and 425 ± 60 [307; 510] μV, respectively. At the same time, the OS a and b wave values were, respectively, 93 ± 33 [52; 171] μV and 407 ± 72 [239; 532] μV before administration; and 97 ± 35 [56; 169] μV and 422 ± 56 [309; 499] μV after administration. No statistically significant differences (*p* < 0.05) were detected between OD and OS before and after the administration of the CS/HA-EPOβ.

To sum up, no statistically significant differences were detected in the ERGs before and after the subconjunctival administration of the nanoparticles or between treated and control eyes. Therefore, the CS/HA nanoformulation did not seem to have a negative influence in the retina, either with or without encapsulated EPOβ.

### 2.4. Histologic Evaluation

The last step of this study was the histologic evaluation of cross-sections of the ocular globes preserved in paraffin blocks. Immunofluorescence was performed to detect if EPOβ had reached the retinal layers while hematoxylin and eosin (HE) staining was used to evaluate the cellular structure.

**Figure 8:**
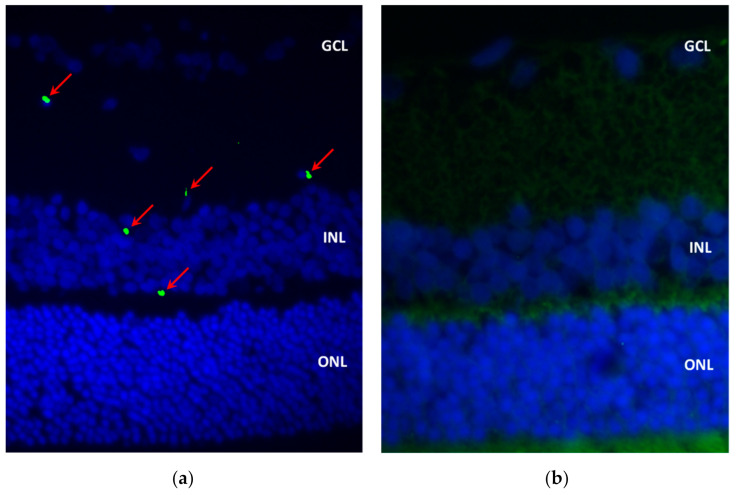

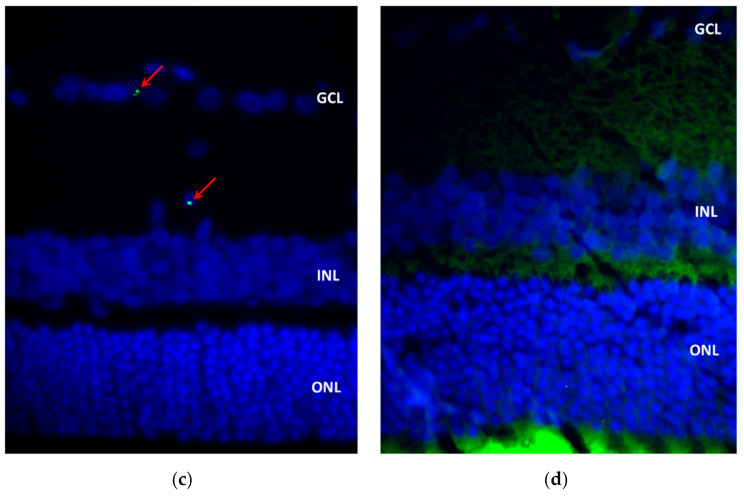
Immunofluorescence image showing a cross-section of the retina after CS/HA-EPOβ administration: (**a**) OD from group A; (**b**) OS from group A (control); (**c**) OD from group F; (**d**) OS from group F (control). Green and blue channels were merged, and green tissue auto-fluorescence is visible in (**b**,**d**). Red arrows indicate EPOβ and cell nuclei are blue (DAPI) (40×). GCL, ganglion cell layer; INL, inner nuclear layer; ONL, outer nuclear layer.

**Figure 9:**
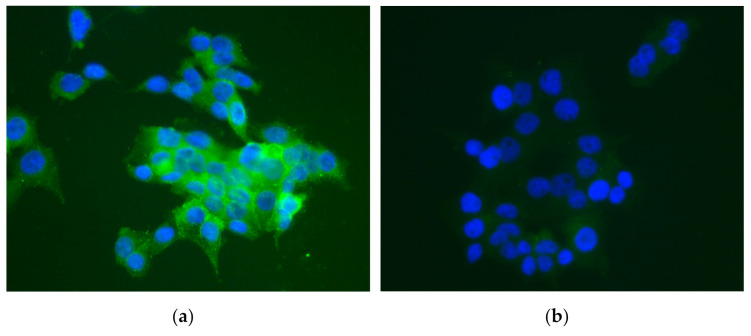
Immunofluorescence image of the HepG2 cells: (**a**) positive control showing EPO in green and nuclei in blue; (**b**) negative control showing a very light green (auto-fluorescence) surrounding the nuclei in blue. Green and blue channels were merged (40×).

Regarding the immunofluorescence findings, 12 h after the administration of the nanoformulation (group A), we detected EPOβ in the corneal endothelium, ciliary body, posterior capsule of the lens, vitreous, sclera, and the retinal ganglion cell layer of the OD. From group B to group F, EPOβ was most commonly detected throughout the retinal cell layers, and secondly in the vitreous, choroid, and sclera, among other tissues. The strength and number of fluorescent signals (EPOβ) decreased with time, and at day 21 (group F), fewer fluorescent dots remained detectable. Figure 8 shows fluorescent signals corresponding to EPOβ distributed throughout the retinal layers. On day 28 (group G), no EPOβ was observed in any ocular tissue. Interestingly, Group E (14 days) revealed an unexpected number of fluorescent signals in the vitreous chamber, in comparison with previous groups, indicating a possible delay in EPOβ absorption from the subconjunctival area. Additionally, EPOβ was not detected in any of the control eyes (OS) of all groups. HepG2 cells served as positive controls (Figure 9) to guarantee the accuracy of the immunofluorescence technique.

**Figure 10:**
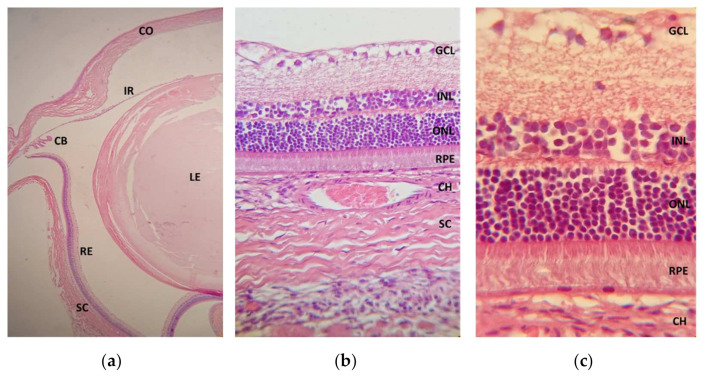
Photomicrographs of the treated eye cross-sections after CS/HA-EPOβ administration stained with hematoxylin and eosin: (**a**) ocular globe from group C (magnification 4×); CO, cornea; IR, iris; CB, ciliary body; LE, lens; RE, retina; SC, sclera. (**b**) cross-section from a rat’s retina from group D (magnification 40×); CH, choroid; GCL, ganglion cell layer; INL, inner nuclear layer; ONL, outer nuclear layer; RPE, retinal pigment epithelium. (**c**) cross-section from a rat’s retina from group E (magnification 100×).

Regarding the cellular structural evaluation through the HE staining, no signs of cellular damage or morphology changes were observed, and no differences were detected between the OD and the OS in any group. Figure 10 represents the microscopic view of OD ocular tissues stained with HE, including the retina, in more detail.

## 3. Discussion

Chitosan and hyaluronic acid are well-known polymers with remarkable mucoadhesive characteristics, along with excellent biocompatibility and versatility in use [15,16,17,18,19,21,22,23]. Our team already described CS/HA nanoparticles carrying EPOβ designed for ocular use, and the empty nanoparticles presented size of 300 ± 6 nm; polydispersity index (PdI) of 0.219 ± 0.043; and zeta potential (ZP) of 33 ± 1 mV. Nanoparticles with 1000 IU of EPOβ were not significantly different from empty nanoparticles in terms of size (289 ± 3 nm), PdI (0.126 ± 0.085), and ZP (39 ± 1 mV), and they were noncytotoxic for both ARPE-19 and HaCaT cells. The CS/HA-EPOβ nanoparticles in vitro release in simulated tear fluid (37 °C, pH 7.4) showed that 60–70% of the EPOβ was released within the first 15 min, followed by sustained release of about 90% within 6 h. Moreover, the drug loading was 17.4 ± 0.1%, and the encapsulation efficiency was 38.4 ± 0.3% [24].

EPOβ, a recombinant human EPO, was chosen as an active principle due to its potential neuroprotective and neuroregenerative qualities, with the perspective of helping in glaucoma treatment. A recent review article describes the antiapoptotic, angiogenic, anti-inflammatory, antioxidant, and neuroprotective effects of EPO in the ocular tissues, including the retina [25]. Subconjunctival administration of EPOβ in solution (NeoRecormon^®^) in rats was already performed by our team, which proved that EPOβ reached the inner layers of the retina with one single dose [8,9,10]. Due to the minor-invasive nature of this route of administration, it was selected to administer CS/HA-EPOβ nanoparticles to rats to assess its safety and ocular penetration in healthy animals.

Considering the results, one day after the administration, 3 rats showed a bilateral discrete reddish discharge in the ocular medial canthus, with no other ocular signs of disease. Chromodacryorrhea is the excessive production of porphyrin-pigmented tears secreted by the harderian gland [26]. It can be due to stress and considering the small amount of discharge presented and the fact that it disappeared after one day, this ocular sign was not considered relevant. Animals presented normal behavior and normal ophthalmological examinations in both eyes. The variations in IOP values after the subconjunctival administration shown in Figure 1 were between the physiological range, according to the literature [27], and no significant differences were observed between the OD and the OS. On day 0, IOP was measured when the animals were still anesthetized, which justifies the lower IOP values when compared with the following time points. These findings denote the high tolerance and safety of the nanoformulation.

It is known that EPO stimulates red blood cell production in bone marrow [2]. Although it was reported that EPOβ did not alter the hematocrit of rats when administered by the subconjunctival route [8], in this study, EPOβ is coated with chitosan and hyaluronic acid, and there were no reports until the moment on how these substances would interact together. Thus, the microhematocrit assessment was necessary to evaluate the systemic impact of subconjunctival CS/HA-EPOβ nanoparticles. No statistically significant differences (*p* < 0.05) were detected in the mean hematocrit values before and after the subconjunctival administration, which was around 45% (Table 1). Although differences in the mean values between groups existed, it was attributed to individual variability. Hematocrit results were within the physiological range, which is between 45 ± 6% and 50 ± 2% for rats with 6 to 12 months old [28], which exclude a significant influence of CS/HA-EPOβ nanoparticles in erythropoiesis.

Flash ERG is the recommended procedure to evaluate retinal function in rats. The recorded waveforms following light stimulation represent a range of cell responses [29]. Photoreceptors’ hyperpolarization is shown as a negative deflection following the flash onset, and it is called an a-wave [30]. Under photopic conditions, a-wave represents cone function, and under scotopic conditions, it indicates rod function [31]. The b-wave is a positive wave that represents the activity of the bipolar and Müller cells [30]. Figure 3, Figure 5 and Figure 7 are good ERG examples retrieved from our assays, where no statistically significant differences were found between treated and control eyes. Although protocols from other studies may differ from ours, both the photopic and the scotopic ERGs recorded in this study have comparable waveforms, in terms of shape and amplitude, to those described in the literature for healthy albino rats [30,32]. The mean a and b waves of the SLR and the SA were comparable to the scotopic ERG results of a study that utilized silver electrodes and the Ganzfeld stimulator [33]. The same study performed light-adapted ERG, and the mean b-waves were similar to our PA results. A-wave amplitudes in the PLR were quite constant while b-waves increased, which matches what is described in the literature, as the a-wave cannot be produced when the stimulus exceeds a certain intensity [34]. Regarding PF, our results showed similarities to those found in the literature [32], which indicates that cone function was preserved. Considering the totality of the ERG results, no influence of the CS/HA-EPOβ nanoparticles was detected in retinal cells electrophysiological responses, which corroborates the local safety of this nanoformulation.

Immunofluorescence was effective in detecting EPOβ in ocular tissues. The fluorescent signal in the treated eye (OD) became gradually weaker and was completely absent at the end of the study (28 days). It is clear that CS/HA nanoparticles administered by the subconjunctival route efficiently delivered EPOβ to the retina. The nanoparticles allowed EPOβ to trespass the outer layers of the ocular globe to reach the inner layers after only 12 h. This fast delivery to the retina might be attributed to the hydrophilic nature of EPOβ, due to its high degree of glycosylation [26], which means that the preferential route of EPOβ absorption would be the conjunctival-scleral pathway, as it is more successful to the posterior segment delivery [35]. In previous studies, EPOβ was also detected in the retina after subconjunctival administration of a commercial solution (NeoRecormon^®^) [8,9]. Moreover, in this study, EPOβ was likewise detected in the corneal endothelium and ciliary body, and the conjunctival-scleral absorption would be unlikely to contribute to this finding due to the presence of the blood-aqueous barrier [36]. Possibly, EPOβ reached the anterior ocular tissues coming from the corneal pathway due to some amount of nanoformulation that overflew from the subconjunctival bubble. This means that EPOβ could diffuse from the CS/HA nanoparticles and trespass the cornea, which is in accordance with our previous ex vivo results using porcine ocular tissues [24]. Another study from our team had already demonstrated ex vivo corneal permeability to EPOβ using a commercial aqueous solution (NeoRecormon^®^) [37].

Another interesting result was that a larger number of fluorescent signals were observed in the vitreous chamber on day 14 compared to previous time points. Considering the high mucoadhesive strength of CS/HA nanoparticles and the corneal permeation due to the formulation’s overflow from the conjunctiva, it would be plausible to deduce that nanoformulation remained in the subconjunctival area and/or in the cornea and enabled a delayed EPOβ permeation. A smooth fluorescent signal was still present at day 21 after the subconjunctival administration, indicating that EPOβ could have a mid-term therapeutic effect with one single dose of 1000 IU. A long-term effect would probably require additional administrations of CS/HA-EPOβ because no EPOβ was detected on day 28 after the administration. As no cellular damage or structural modifications were detected by observing ocular cross-sections stained with HE, and no differences were observed between test and control eyes, the safety of the CS/HA nanoparticles with and without EPOβ is sustained, as already verified in our previous in vitro studies [24]. Besides, it also indicates that EPOβ is safe when administered by the subconjunctival route using this formulation. The safety of the subconjunctival sole administration of EPO solution has already been demonstrated [8,9].

In conclusion, CS/HA nanoparticles are an innovative ocular delivery system for EPOβ that was considered biologically safe since they did not cause any local or systemic adverse side effects. These nanoparticles allowed for a sustained EPOβ retinal delivery during 21 days using a minor invasive route of administration. This nanoformulation could target neuroprotection in cases of retinal diseases such as glaucoma and other retinopathies. Further research to assess EPOβ pharmacokinetics and pharmacodynamics after ocular administration should also be considered.

## 4. Materials and Methods

### 4.1. Materials

Adult male Wistar Hannover albino rats (*n* = 21) were the model selected for this study, with an average weight of 317 ± 36 g. Animals were acquired from Charles River Laboratories (Saint-Germain-Nuelles, France). Chitosan of low molecular weight (LMW CS, 100 kDa, 92% deacetylation) was obtained from Sigma Aldrich (Irvin, UK). The sodium hyaluronate eyedrop grade quality (300 kDa—Eye) from Shandong Topscience was a kind gift from Inquiaroma (Barcelona, Spain). The recombinant human erythropoietin (epoetin beta, EPOβ) used was NeoRecormon^®^ 30000 IU (RocheDiagnostics GmbH, Mannheim, Germany). The subconjunctival administration was performed using insulin syringes with 29 G needles (Becton Dickinson^®^ Micro-Fine Insulin Syringe 0.5 mL). The drugs used were ketamine (Ketamidor^®^ 100 mg/mL, Richter Pharma, Wels, Austria), medetomidine (Domtor^®^ 1mg/mL, Orion Corporation, Espoo, Finland), atipamezole (Antisedan^®^ 5 mg/mL, Zoetis, Parsippany-Troy Hills, NJ, USA) and sodium pentobarbital (Euthasol^®^ 400 mg/mL, Animalcare Group, North Yorkshire, UK) available in the Faculty of Veterinary Medicine (ULisboa). The ERG equipment (RETIcom, Roland Consult, Stasche & Finger GmbH, Brandenburg, Germany), the rebound tonometer (Tonolab^®^, Icare, Finland), the slit lamp (Hawk Eye^®^, Dioptrix, France), and the PanOptic^®^ ophthalmoscope (WelchAllyn-Hillrom, Chicago, IL, USA) belonged to the Faculty of Veterinary Medicine (ULisboa). The positively charged microscope slides used were Epredia™ SuperFrost Plus™ Adhesion slides (ThermoFisher Scientific, Waltham, MA, USA). Cover plates and immunostaining rack (Epredia™ Shandon™ Sequenza™, ThermoFisher Scientific, Waltham, MA, USA) and Thermo Scientific™ Gemini™ AS (Waltham, MA, USA) slide stainer for hematoxylin and eosin stain belonged to the Faculty of Veterinary Medicine (ULisboa). HepG2 human-derived liver hepatocellular carcinoma cell line (ATCC^®^ HB-8065™). All the cell culture media and supplements were from Gibco (ThermoFisher Scientific, Waltham, MA, USA). UltraCruz^®^ Blocking Reagent (sc-516214) was from Santa Cruz Biotechnology (Dallas, TX, USA). EPO monoclonal primary antibody 4F11 (MA5-15684) and goat anti-mouse IgG (H+L) secondary antibody DyLight 488 (35502) were from Invitrogen (ThermoFisher Scientific, Waltham, MA, USA). The mounting medium used was UltraCruz^®^ Aqueous Mounting Medium with DAPI (sc-2494; Santa Cruz Biotechnology, Dallas, TX, USA). Axioscop 40 fluorescence microscope with an Axiocam HRc camera (Carl Zeiss, Germany) and the Zetasizer Nanoseries Nano S and Nano Z (Malvern Instruments, Malvern, UK).

### 4.2. Methods

#### 4.2.1. Animals and Timepoints

Wistar Hannover rats (*n* = 21) were split into 7 groups (*n* = 3). Each group of rats were housed in a type IV cage (1875 cm^2^ of floor area) with a stainless-steel wire cover. Food pellets and water were offered *ad libitum*, and the room environment was maintained in a 12 h cycle of light/ darkness, at 20 ± 2 °C and an average humidity of 50–60%.

Each group corresponded to a specific time point as follows: Group A (*n* = 3) corresponding to 12 h after the injection; Group B (*n* = 3) to 1 day; Group C (*n* = 3) to 3 days; Group D (*n* = 3) to 7 days; Group E (*n* = 3) to 14 days; Group F (*n* = 3) to 21 days; and finally, Group G (*n* = 3) corresponding to 28 days. These specific time points correspond to the time of euthanasia after the subconjunctival administration of CS/HA-EPOβ nanoparticles. This study was performed in accordance with animal ethical requirements, and it was approved by the Organ Responsible for Animal Welfare (Órgão Responsável pelo Bem-Estar dos Animais—ORBEA) of the Faculty of Veterinary Medicine, University of Lisbon, and by the national entity General Directorate of Food and Veterinary (Direção Geral de Alimentação e Veterinária—DGAV).

#### 4.2.2. Ophthalmological Examination

**Figure 11:**
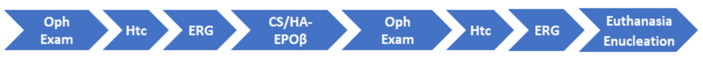
Sequence of procedures performed in each animal (*n* = 21). Oph Exam–Ophthalmological examination; Htc–blood collection for microhematocrit; ERG–electroretinography; CS/HA-EPOβ–subconjunctival administration of the nanoformulation.

Figure 11 summarizes the occurrence of the events. All animals (*n* = 21) underwent a complete ophthalmological examination prior to being included in the study, including measurement of the intraocular pressure with a rebound tonometer (Tonolab^®^, Icare, Finland), biomicroscopic examination of the anterior segment with a slit lamp (Hawk Eye^®^, Dioptrix, France), and posterior segment examination with PanOptic^®^ ophthalmoscope (WelchAllyn-Hillrom, Skaneateles Falls, NY, USA). Complete ophthalmological examinations were then performed periodically after the subconjunctival administration of CS/HA-EPOβ nanoparticles: 1 h, 12 h and 1, 2, 3, 7, 14, 21, and 28 days after.

#### 4.2.3. Electroretinography

**Figure 12:**
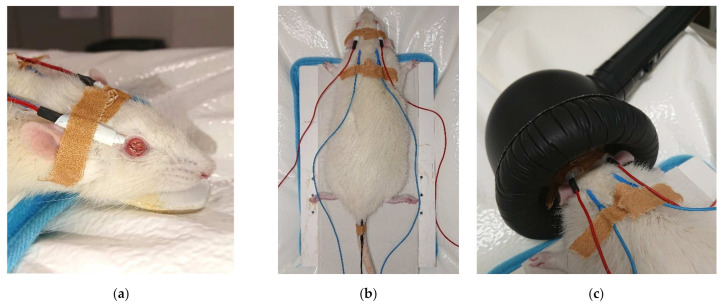
Pictures the ERG set: (**a**) active electrodes (red) with silver tips placed on corneas; (**b**) reference electrodes (blue) placed next to the ears and the ground electrode (black) at the base of the tail; (**c**) head positioning inside the MiniGanzfeld stimulator.

Flash electroretinography (ERG) was performed in all animals at two periods of time: the first ERG preceded the administration of CS/HA-EPOβ nanoparticles, and the second ERG preceded euthanasia. The ERG procedure was based on previously published protocols [13] in which rods and cones activity were recorded as a-wave and b-wave amplitudes (μV) in response to luminous stimuli. Animals were kept in the dark for 12 h before the exam. General anesthesia was required for a total immobilization of the animal. A combination of ketamine (70 mg/kg) and medetomidine (0.8 mg/kg) was administered intraperitoneally to induce anesthesia. Active electrodes with wired silver tips were placed on both corneas (Figure 12a) after one drop of oxybuprocaine hydrochloride (Anestocil^®^) followed by one drop of a carbomer-based gel (Lubrithal^®^). Reference electrodes were placed subcutaneously, and the tip of the electrode was located between the ear and lateral cantus of both sides, and the ground electrode was placed at the base of the tail (Figure 12b). A heating pad was used to maintain the animal’s body temperature, which was periodically checked. For light stimulation, a MiniGanzfeld was placed on the animal’s head (Figure 12c). Signals were recorded independently for each eye and the impedance < 5 kohms at 0.1–1000 Hz frequency (0 dB = 3 cds/m^2^). Each ERG examination lasted 75 min and was divided into 5 parts. The first part was the scotopic luminance response (SLR) which consisted of 9 intensities of light flashes from –35 dB to +5 dB, delivered three times at 0.1 Hz. The next part was the photopic adaptation (PA), in which the flashes intensity was calculated at the maximum b-wave amplitude of the SLR. Flashes were delivered three times at 1.3 Hz at 0, 2, 4, 8, and 16 min of light adaptation. Then, the photopic luminance response (PLR) used light flashes of 9 intensities from –35 dB to +5 dB, delivered three times at 1.3 Hz. After 10 min of light adaptation, the photopic flicker delivered flashes of 0, –5, –10, and –15 dB at 6.3 Hz. Lastly, the scotopic adaptation (SA) used white dim flashes delivered three times at 1.3 Hz at 0, 2, 4, 8, 16, and 32 min of dark adaptation. Rod function was tested by stimulating the retina with dim flashes in scotopic conditions (–35 dB = –3.02 log cds/m^2^), while cone function was assessed by retinal stimulation with bright flash’s (+5 dB = 0.98 log cds/m^2^) and 6.3 Hz flicker in photopic conditions.

#### 4.2.4. Hematocrit

A blood sample was retrieved from each animal during the anesthesia for the ERG to assess microhematocrits. The rat tail was immersed in warm water (≈40 °C) for 3 to 5 min, and the blood was collected from a lateral vein by a needle punch (23 G needle) with the aid of a capillary tube. Samples were centrifuged in a capillary centrifuge at 10,000 rpm for 5 min, and microhematocrits were read using a proper scale. This procedure was performed twice before the first ERG and before the second ERG.

#### 4.2.5. Nanoparticles’ Preparation

The formulation was prepared on the day of the experimental procedures, based on previously published methods [22,23] which consist of a modified ionotropic gelation technique. All reagents were previously sterilized using a 0.22 µm sterile filter in a laminar flow cabinet, and the nanoparticles were produced with EPOβ (CS/HA-EPOβ nanoparticles; 1000 IU of EPOβ), and without EPOβ (empty CS/HA nanoparticles) in a laminar flow cabinet following the previously published procedure [24]. Firstly, the chitosan solution at 2 mg/mL in 0.1% (*v*/*v*) of acetic acid was diluted in NaCl 0.9% in 1:1 (*v*/*v*) ratio (CS-NaCl; 1 mg/mL). Separately, the hyaluronic acid solution at 2 mg/mL in purified water was mixed with 1000 IU of EPOβ (NeoRecormon^®^) at a 1:1 (*v*/*v*) ratio (HA-EPOβ; 1 mg/mL). Finally, HA-EPOβ was slowly added to CS-NaCl, and the formation of the nanoparticles occurred instantly by self-assembly. For the empty CS/HA formulation (without EPOβ), the same volume of purified water was added to the hyaluronic acid instead of EPOβ. The formulation was aspirated with a sterilized insulin syringe to be further administered subconjunctivally.

#### 4.2.6. Subconjunctival Administration

**Figure 13:**
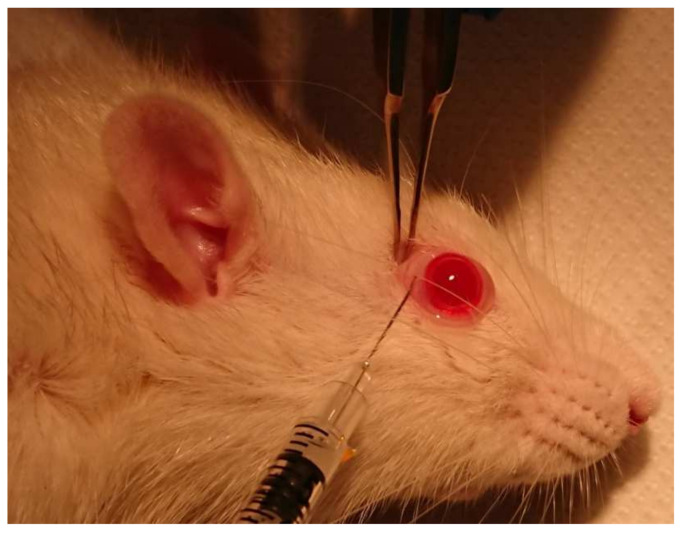
Photo of the subconjunctival administration of the C/HA-EPOβ in the OD of a rat, under general anesthesia.

At the end of the first ERG, during anesthesia, all animals underwent subconjunctival administration of 80 µL of CS/HA-EPOβ nanoparticles (1000 IU of EPOβ) in the right eye (OD); and 80 µL of empty CS/HA nanoparticles (without EPOβ) were administered in the left eye (OS), which served as a negative control. The subconjunctival administration (Figure 13) was performed with an insulin syringe with a 29 G needle at 3 different subconjunctival sites, using a binocular magnifier. Anesthesia was reverted with subcutaneous atipamezole (2.5 mg/kg).

#### 4.2.7. Euthanasia and Enucleation

At 12 h (*n* = 3), 1 day (*n* = 3), 3 days (*n* = 3), 7 days (*n* = 3), 14 days (*n* = 3), 21 days (*n* = 3) and 28 days (*n* = 3) after the administration of the CS/HA-EPOβ formulation, euthanasia was performed with intraperitoneal sodium pentobarbital (150 mg/kg). Before euthanasia, a second blood sampling for microhematocrit evaluation and a second ERG was performed.

**Figure 14:**
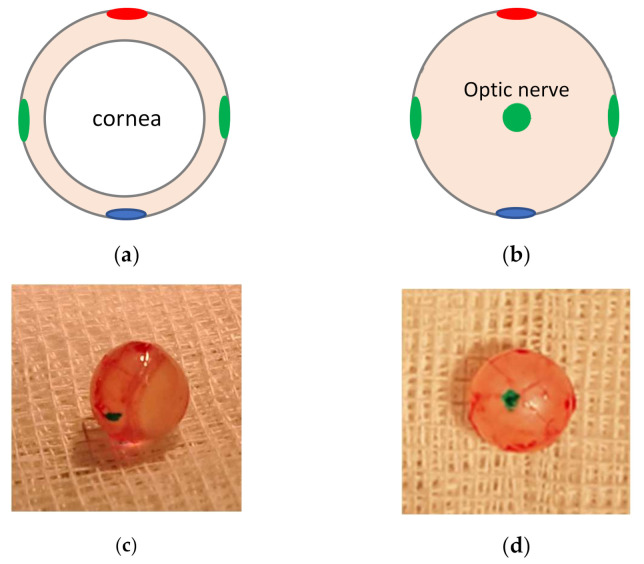
Representation of a rat ocular globe painted with tissue dyes: (**a**) frontal view; (**b**) caudal view. Photo of a rat ocular globe painted with tissue dyes: (**c**) lateral view; (**d**) caudal view, in green we can identify the transected optic nerve.

Immediately after euthanasia, both eyes were enucleated and painted with tissue dyes in the optic nerve and the lateral, medial, dorsal, and ventral sides, as shown in Figure 14, to allow for correct orientation of the histological sections. Eyes were stored in 10% (*v*/*v*) formaldehyde in PBS (0.1 M, pH 7.4) and further included in paraffin blocks.

#### 4.2.8. Histologic Evaluation

After including the ocular globes in paraffin blocks, immunofluorescence and hematoxylin and eosin (HE) stains were performed. From the OD and the OS, cross-sections of 3 μm each were made using a microtome, 4 cross-sections per eye were used for immunofluorescence, and 4 cross-sections per eye were used for HE staining. For immunofluorescence, sections were placed in adhesion slides and deparaffinized in xylol for 15′ + 5′, rehydrated in alcohol from 100° to 70° (3′ each) and purified water (3′ + 15′). A sequence of washing steps with Triton X-100 solution (0.1% *v*/*v* in PBS) and Tween 20 solution (0.1% *v*/*v* in PBS) was performed, followed by assembling of the slides in cover plates. Sections were incubated with UltraCruz^®^ Blocking Reagent at room temperature for 60′. Then, EPO monoclonal primary antibody 4F11 (1:400) was added, followed by overnight incubation at 4 °C. On the following day, cross-sections were washed with Tween 20 solution (0.1% *v*/*v* in PBS) and incubated with goat anti-mouse secondary antibody DyLight 488 (1:1000) in the dark, at room temperature, for 60′. Cross-sections were washed again in PBS, and the slides were carefully disassembled from the cover plates. A small amount of UltraCruz^®^ mounting medium with DAPI was added in each slide, and a coverslip was placed on the top, followed by sealing with varnish. Sections were analyzed using an Axioscop 40 fluorescence microscope with an Axiocam HRc camera (Carl Zeiss, Germany). The emitted fluorescence was observed and recorded in images processed with AxioVision software (Rel.4.8.1, Carl Zeiss). Negative controls were the OS, and the positive controls were HepG2 cell cultures because they naturally express EPO. These cell cultures were submitted to a hypoxic environment for 120′ at 37 °C and then fixed in 10% (*v*/*v*) formaldehyde in PBS for 15′ at room temperature in the dark. HepG2 cells were washed with Triton X-100 solution (0.1% *v*/*v* in PBS) for 5′. From this step on, the protocol was the same as the cross-sections. For HE, all sections were placed in regular slides and processed in the multi-tasking stainer Gemini™ AS.

#### 4.2.9. Statistical Methods

The experimental data were statistically assessed using GraphPad Prism version 6.0 (GraphPad Software, San Diego, CA, USA) and Microsoft Office Excel (Microsoft, Albuquerque, NM, USA) by one-way ANOVA and *t*-test to detect significant differences between means. The established statistical significance was 95%, corresponding to a *p*-value of 0.05. Data were presented as mean ± standard deviation (SD).

## Figures and Tables

**Table 1:** Each group’s hematocrit (Htc) was obtained from the microhematocrit measurement before and after the CS/HA-EPOβ nanoparticles administration (mean ± SD; %). After–before euthanasia.

		Group A (12 h)	Group B (1 day)	Group C (3 days)	Group D (7 days)	Group E (14 days)	Group F (21 days)	Group G (28 days)
Htc (%)	Before	42.0 ± 1.0	48.0 ± 2.6	47.0 ± 1.7	44.0 ± 2.6	42.7 ± 2.5	42.3 ± 2.5	45.5 ± 2.6
	After	42.7 ± 1.5	49.0 ± 2.6	47.7 ± 3.2	44.3 ± 4.0	42.7 ± 2.1	42.3 ± 2.5	45.4 ± 2.8

**Table 2:** Photopic adaptation-mean amplitudes of the a-wave and the b-wave (µV) recorded from the OD and OS in response to light-stimuli at 0, 2, 4, 8, and 16 min of light adaptation. Values correspond to before and after the administration of the nanoparticles (after–before euthanasia). Data represent all groups.

		a-Wave (µV)		b-Wave (µV)	
	min	OD	OS	OD	OS
Before	0	19 ± 12	16 ± 13	212 ± 36	211 ± 59
	2	20 ± 16	20 ± 13	201 ± 36	193 ± 43
	4	13 ± 12	14 ± 11	198 ± 50	199 ± 44
	8	18 ± 16	17 ± 12	202 ± 46	214 ± 53
	16	16 ± 12	19 ± 14	200 ± 40	201 ± 44
After	0	19 ± 18	21 ± 18	216 ± 45	213 ± 52
	2	20 ± 14	19 ± 12	204 ± 38	193 ± 41
	4	14 ± 11	20 ± 18	214 ± 48	192 ± 37
	8	16 ± 9	16 ± 14	208 ± 42	202 ± 47
	16	16 ± 11	18 ± 12	212 ± 44	201 ± 46
